# Comparing progesterone in blubber and serum to assess pregnancy in wild beluga whales (*Delphinapterus leucas*)

**DOI:** 10.1093/conphys/coz071

**Published:** 2019-11-11

**Authors:** Caroline E C Goertz, Kathy Burek-Huntington, Katie Royer, Lori Quakenbush, Tonya Clauss, Roderick Hobbs, Nicholas M Kellar

**Affiliations:** 1 Alaska Sea Life Center, 301 Railway, Seward, AK 99664, USA; 2 Alaska Veterinary Pathology Service, 23834 The Clearing Dr, Eagle River, AK 99577, USA; 3 Alaska Department of Fish and Game, Fairbanks, AK 99701, USA; 4 Georgia Aquarium, Atlanta, GA 30313, USA; 5 Marine Mammal Laboratory, National Marine Fisheries Service, Nationa Oceanic and Atmospheric Administration, Seattle, WA 98115; 6130 NE 204th St., Kenmore, WA 98028, USA; 6 Marine Mammal and Turtle Division, Southwest Fisheries Science Center, National Marine Fisheries Service, National Oceanic and Atmospheric Administration, LaJolla, CA 92037 USA

## Abstract

The Cook Inlet population of beluga whales (*Delphinapterus leucas*) is listed as endangered and continues to decline for largely unknown reasons; however, there is some evidence that poor reproductive success is a contributing factor. Pregnancy is difficult to detect through observation, and, there is reluctance to capture endangered beluga whales for reproductive tract imaging via ultrasound or to obtain suitable samples for pregnancy assessments. An endocrine analysis of blubber biopsies collected by remote darting could represent a minimally invasive way to identify pregnant females and compare pregnancy rates among years or populations. Studies have validated the use of blubber biopsies to identify pregnant females in other cetacean species, but not beluga whales; therefore, validation of blubber progesterone levels to proven tests that reliably detect pregnancy was needed for this species. As part of a larger study, we sampled blood and blubber from live-captured beluga whales (21 females, 26 males) in Bristol Bay, Alaska. Progesterone levels were determined in serum samples obtained from all animals and in blubber samples from a subset (14 females, 13 males) to determine pregnancy status, estimate the stage of pregnancy, and evaluate the suitability of using blubber alone for these assessments. In general, there was distinct separation of high levels of progesterone in serum and blubber for presumed pregnant females and low levels for males and presumed non-pregnant females. Blubber progesterone levels in two females (14% of females tested) were intermediate (i.e. ambiguous); their corresponding serum levels were consistent with being pregnant in one case and not being pregnant in the other. Except for these two intermediate values, pregnancy status of beluga whales could be determined from blubber alone, thereby providing a valuable tool to better understand reproduction dynamics from populations that cannot be captured for examination.

## Teaser

Blood and blubber were sampled from live-captured beluga whales in Bristol Bay, Alaska. Progesterone levels were used to assess pregnancy status. For most cases, blubber progesterone levels correlated with serum, indicating that pregnancy can be assessed using a remote biopsy and thereby providing a valuable tool to better understand reproduction dynamics from populations that cannot be readily captured for examination.

## Introduction

The Cook Inlet, Alaska population of beluga whales (*Delphinapterus leucas*) is listed as endangered and is not recovering. While the reason for the lack of recovery is unknown, poor reproductive success was documented in 6 of 7 years of monitoring calf production (Hobbs *et al.*, 2015) and abortions have been detected in dead stranded animals with an apparent increase in 2008 (Burek-Huntington *et al.*, 2015). The stage, or stages, at which reproductive failure is occurring is unknown. Understanding pregnancy rate and frequency and timing of failure in endangered populations is important to identify impediments to recruitment, evaluate recovery options, and project population growth. Pregnancy is commonly determined by measuring progesterone in blood or urine. In captive populations, pregnancy is also monitored using reproductive ultrasound examinations to confirm the presence and size of the fetus. There is reluctance to capture animals from listed populations to perform ultrasound and collect biological samples due to the disturbance related to capture and handling and the logistics of collecting sufficient sample sizes. Pregnancy can be difficult to detect and monitor through observation of free-swimming cetaceans; therefore, studies of wild marine mammals would benefit from the use of samples obtained without capturing individuals.

Hormones have been analyzed in fecal samples collected from the water for some large whales (Corkeron *et al.*, 2017; Wasser *et al.*, 2017). Unfortunately, beluga feces tend to disperse rapidly, making them difficult to collect and likely unsuitable for analysis. Hormones present in blow has been collected from large whales (Hogg *et al.*, 2009) and trained beluga whales (Richard *et al.*, 2017). However, such sampling has not yet been attempted with wild beluga whales and may not be practical for that species due to their surfacing and blow patterns. Beluga blow is lower volume than the exhalations from larger whales, requiring sampling devices to be closer to the whales increasing disturbance and potential risk of collision. Additionally, beluga surfacing dynamics are less predictable, especially during close approaches. Their short surface duration, irregular breathing intervals, and small target area often in turbid water likely make it difficult to adequately position collection devices; however, this technique may be useful for sampling groups of live-stranded whales. Studies have validated the use of blubber biopsies to identify pregnancy in many species of wild cetaceans, including *Balaenaptera acutorostrata* (Mansour *et al.*, 2002)*, Megaptera novaeangliae* (Pallin *et al.*, 2018b)*, Delphinus capensis* (Trego *et al.*, 2013)*, Delphinus delphis* (Kellar *et al.*, 2006)*, Globicephala melas* (Pérez *et al.*, 2011)*, Lagenorhynchus obliquidens* (Kellar *et al.*, 2006)*, Lissodelphis borealis* (Kellar *et al.*, 2006)*, Phocoenoides dalli* (Trego *et al.*, 2013)*, Stenella attenuata* (Trego *et al.*, 2013)*, S. longirostris* (Trego *et al.*, 2013) and *Tursiops truncatus* (Kellar *et al.*, 2017; Pérez *et al.*, 2011). The use of remotely deployed biopsy darts would be a minimally invasive way to identify pregnant females in the Cook Inlet beluga whale population and compare pregnancy rates with other populations; however, interpreting the results requires understanding the relationship between blubber progesterone and other tests known to detect pregnancy.

The timing of calving varies among populations and occurs between April and October. (Brodie, 1971; Burns and Seaman, 1986; Suydam, 2009). The beluga gestation period is an average of 473 days (range, 444–507) or 15.6 months (Robeck *et al.*, 2015); therefore, breeding occurs the previous year between January and July. Timing of birth also varies within populations; neonates have been observed in Cook Inlet, as early as April (Huntington, 2000) and as late as October (McGuire *et al.*, 2016). In our study area, Bristol Bay, most births occur in May and June (Burns and Seaman, 1986). Because gestation is longer than a year, there is a period within each year when “pregnant” whales include females in early gestational and near term stages. Being able to identify the stage of pregnancy during the overlap period is necessary to differentiate annual pregnancy rates from annual birth rates.

As part of a larger study, we sampled blood and blubber from live-captured beluga whales (21 females, 26 males) in Bristol Bay. Progesterone levels were measured in serum samples collected from all animals and in blubber samples from a subset (14 females, 13 males) to determine pregnancy status, estimate the stage of pregnancy and evaluate the suitability of solely using blubber for these assessments.

## Methods

### Capture

Individual whales were captured in the Nushagak arm of Bristol Bay near Dillingham, Alaska in May 2008 (‘spring’ samples) and in September 2008 and 2012, and August 2013 and 2014 (‘fall’ samples). Beluga whales less than 250 cm long and mother–calf pairs were avoided due to research permit limitations. An 18-ft aluminum skiff with a 70-hp outboard was used to follow an individual whale until it was in shallow water, less than 2-m deep, then a buoy attached to a net measuring 125 m long × 4 m deep with a 0.3-m square mesh was dropped and picked up by another boat. Both boats moved towards shore pulling the net, additional boats helped to keep the whale corralled. After capture, staff secured the whale with a tail rope, removed the net, and moved the beluga whale to water shallow enough to allow access for examination, but deep enough to support some of the animal's weight. Hoop nets, slings, and staff provided additional restraint as needed.

### Sampling

Processing of animals included physical examination, measurements, biological sampling, satellite tagging and release. Whales were held for less than 2 hours. Physical examination included visual observations and determination of sex by palpating or, when possible, visual assessment of the urogenital slit. Reproductive ultrasounds were not performed due to limitations of field conditions, equipment and time. Blood was drawn as soon as possible after capture from the periarterial venous rete on the dorsal fluke using 1.3 cm, 19 gauge butterfly catheter (Becton, Dickinson, and Co., Franklin Lakes, New Jersey, USA) after the skin was disinfected with alcohol. Blood was collected for progesterone analysis into plain serum tubes (BD Vacutainer®; Becton, Dickinson, and Co.). Tubes were placed immediately into a cooler with ice packs, later refrigerated, and processed within 12 hours. Aliquots of serum were placed into sterile 2 mL polypropylene cryovials (Fisher Scientific, Pittsburgh, Pennsylvania, USA), frozen on dry ice while in the field, then transferred to an ultralow freezer (−80 °C) until shipped for analysis. Blubber biopsies, 8 mm in diameter and less than 2 cm deep, were taken along the flank below the dorsal ridge in an area accessible for sampling by darting biopsy projectiles using sterile biopsy punches and trochars after cleaning the skin with alcohol. Biopsies were transferred to storage containers and placed on dry ice in the field and later transferred to a liquid nitrogen chilled dry shipper, and ultimately an ultralow (−80 °C) freezer until shipped for analysis.

### Serum analysis

Serum progesterone analysis was determined for all animals at the Diagnostic Endocrinology Laboratory within the Animal Health Diagnostic Center, College of Veterinary Medicine, Cornell University (n = 47, 21 females, 26 males). Concentrations were determined by solid-phase ^125^I radioimmunoassays using commercially available kits (Siemens Healthcare Diagnostics, Los Angeles, CA) run in duplicate. All assays that included samples from beluga whales met all quality assurance criteria, based on manufacturer and internal controls (canine, equine, feline, bovine samples with previously determined levels of progesterone) that were run in each assay. Females with values greater than 6.0 ng/mL were presumed to be pregnant (Calle *et al.*, 1993) which is consistent with other cetacean species (Sawyer-Steffan *et al.*, 1983, data for *Tursiops truncatus*).

### Blubber analysis

Blubber progesterone was determined for a subset of animals by Southwest Fisheries Science Center (Kellar), National Marine Fisheries Service in La Jolla, CA, following a modified protocol (Kellar *et al.*, 2006). Blubber samples did not include the epidermis and were approximately 50 to 150 mg in weight. Samples were homogenized in 1.4 mL 100% ethanol and processed in stainless steel microvials (BioSpec Products, cat. no. 2007) for six 50-second cycles at a speed of 5 m/s (Omni International Inc., Bead Ruptor 24). The supernatant was collected into 13 × 100 mm disposable borosilicate glass culture tubes, and an additional 1.5 mL of 100% ethanol was used to rinse the stainless-steel tubes. Two milliliters of 4:1 ethanol:acetone was added, mixed with a multi-tube vortex (Fisher Scientific MultiTube vortexer, cat. no. 02-215-450) and then centrifuged at 5000 rpm for 15 minutes. The supernatant was transferred to 12 × 75 mm disposable borosilicate glass culture tubes and evaporated under compressed air with an Evap-O-Rac (Cole-Palmer, EW-01610-15) while being incubated in 25 °C water. Two milliliters of diethyl ether were then added. The samples were again vortexed in the multi-tube mixer and centrifuged as above. The supernatant was transferred to new 12 × 75 mm glass tubes, evaporated and following evaporation 1.5 mL acetonitrile was added. The samples were vortexed for 5 minutes and then 1.5 mL hexane was added, forming two immiscible layers. Following another 5 minutes of vortexing, the samples were centrifuged for 15 minutes and placed in a −20 °C freezer for at least 1 hour. The acetonitrile layer was collected and an additional 1.5 mL hexane was added. The samples were vortexed for 5 minutes, centrifuged for 15 minutes, and placed back in the −20 °C freezer for at least 1 hour once more. The acetonitrile layer was collected and then evaporated using compressed air. Residues were resuspended in 250 μL phosphate-buffered saline with 0.1% bovine serum albumin, and mixed by vortex for 15 minutes, and frozen at −20 °C. A commercially available enzyme immunoassay kit (Enzo Life Sciences Inc., ADI-901-011) was used to measure progesterone levels. The standard curve ranged from 15.62 to 500 pg/mL. The estimate interassay coefficient of variation (COV) had a range of 8.3% to 15.1%. The intraassay COV had a range of 6.1–12.9%. Pregnancy status was determined by comparing the individual’s serum progesterone to values seen in pregnant beluga in aquaria (Calle *et al.*, 1993) and blubber progesterone levels were compared with published values from small Delphinids (Kellar *et al.*, 2006; Kellar 2013b; Trego *et al.*, 2013).

### Statistical analysis

Summary statistics (mean and SD) for serum and blubber progesterone were calculated using Excel. Beluga whales with serum progesterone values < 0.02 ng/mL and > 40 ng/mL were assigned the values of 0.02 ng/mL and 40 ng/mL, respectively, for statistical analysis and graphical representation. The combined serum and blubber data were fit to a first-order model using linear least-squares regression in MATLAB (MathWorks, 2018a). The significance of seasonal variation (May vs August/September) in serum or blubber progesterone was assessed using the Mann–Whitney *U* test (http://in-silico.online/).

**Table 1:** Ranges, means, and standard deviations for blubber and serum progesterone (tP4) results from beluga whales live-captured and sampled during 2008–2014. Pregnancy status assigned based on the specific sample tested. Belugas with progesterone values < 0.02 ng/mL and > 40 ng/mL were assigned values of 0.02 ng/mL and 40 ng/mL, respectively

		Pregnancy Status	N May	N Aug/Sep	N Total	Min	Max	Mean	SD
Females	Serum tP4 (ng/mL)	Pregnant	5	8	13^1^	10.85	>40.0	31.18	9.67
	Serum tP4 (ng/mL)	Not Pregnant	3	5	8	0.1	0.71	0.30	0.24
	Blubber tP4 (ng/g)	Pregnant	4	6	10	347.32	656.21	485.53	104.52
	Blubber tP4 (ng/g)	Not Pregnant	0	2	2	0.24	1.33	0.79	0.77
	Blubber tP4 (ng/g)	Uncertain	1^NP-S^	1^P-S^	2	25.37	44.3	34.84	13.39
Males	Serum tP4 (ng/mL)	NA	2	24	26^4^	<0.02	0.23	0.12	0.09
	Blubber tP4 (ng/g)	NA	0	13	13	0.06	0.88	0.32	0.26

^1^Four of these were reported as > 40 ng/mL and limited to 40 ng/mL for statistical analysis

^4^Six of these were reported as < 0.02 ng/mL and limited to 0.02 ng/mL for statistical analysis

^NP-S^Serum tP4 level consistent with not being pregnant

^P-S^Serum tP4 level consistent with being pregnant

tP4 = total progesterone

**Table 2:** Seasonal mean and standard deviations of serum and blubber progesterone from female beluga whales in May and September (2008–2014). P = Pregnant, NP = Not Pregnant. Belugas reported to have serum values > 40 ng/mL were assigned the value of 40 ng/mL. The two females with intermediate blubber values were not included in the blubber calculations. Blubber was not available from non-pregnant females sampled in May. Serum progesterone values from pregnant belugas sampled in May were found to be greater than pregnant belugas sampled in Aug/Sep (p = 0.0318) by the Mann-Whitney U test. No significant difference was found between blubber progesterone sampled from pregnant belugas in different seasons

Month	Pregnancy status	Serum tP4 mean ± sd (ng/mL)	Serum N Total	Blubber tP4 mean ± sd (ng/g)	Blubber N Total
May	P	35.38 ± 10.34	5^1^	505.04 ± 111.91	n = 4
Aug/Sep	P	28.55 ± 8.88	8	472.52 ± 107.9	n = 6
May	NP	0.22 ± 0.17	3	NA	n = 0
Aug/Sep	NP	0.34 ± 0.29	5	0.79 ± 0.77	n = 2

^1^Four of these were reported as > 40 ng/mL but limited to 40 ng/mL for statistical analysis

tP4 = total progesterone

## Results

### Serum results

**Figure 1:**
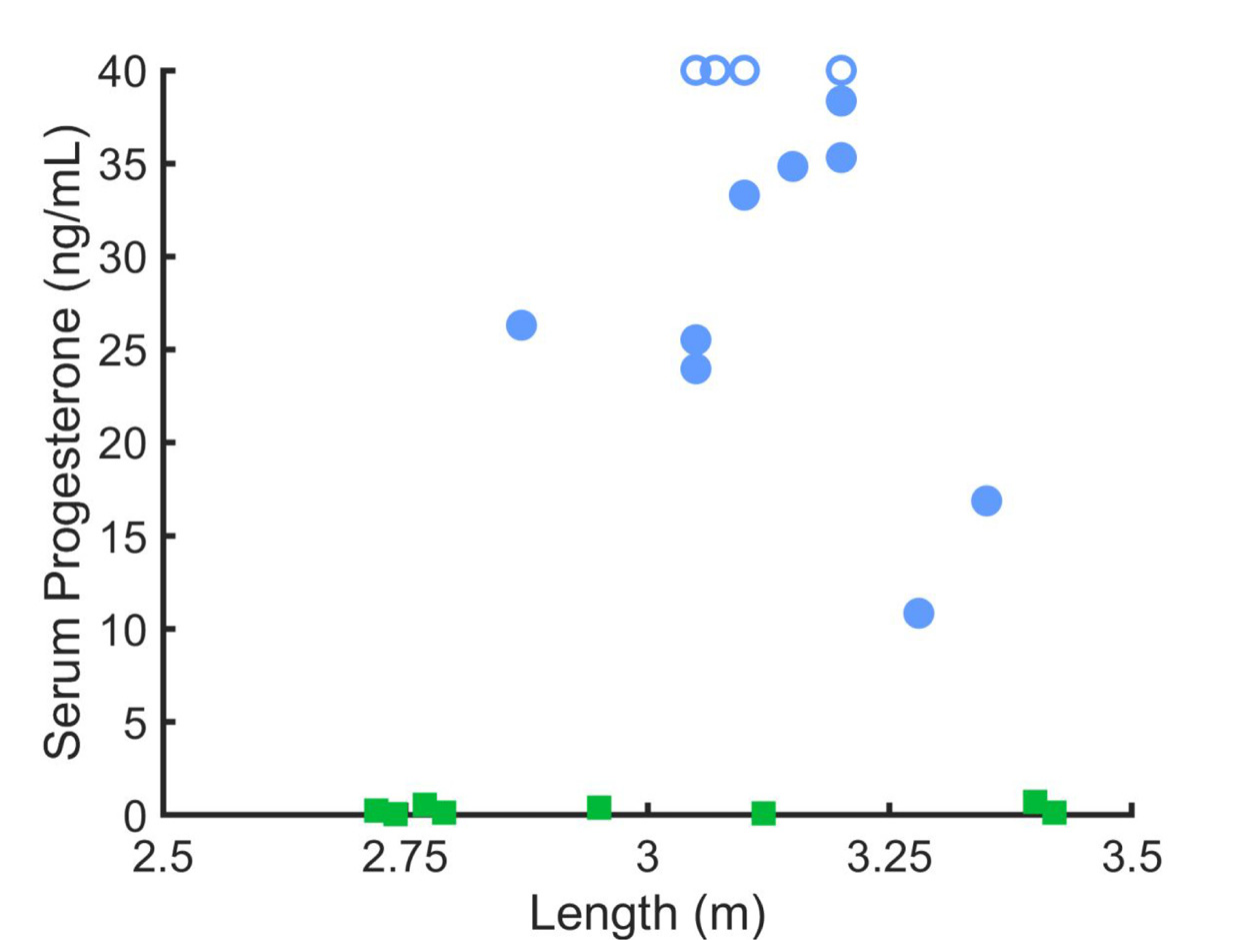
Serum progesterone concentrations in female beluga whales; non-pregnant (green squares, n = 8) and pregnant (blue circles, n = 13) by length of beluga sampled in Bristol Bay (2008–2014). Belugas with serum progesterone values > 40 ng/mL (n = 4) were assigned the value 40 ng/mL (open symbols).

Forty-seven beluga whales, 21 females and 26 males, were live-captured in May, August or September 2008 to 2014. Summary statistics are presented in Table 1. Males (n = 26) had consistently low to non-detectable levels of progesterone ranging from < 0.02 to 0.23 ng/mL with a mean of 0.12 ± 0.09 ng/mL. Values for females (n = 21) were variable but clustered. Females considered pregnant (n = 13) had serum progesterone levels from 10.85 to > 40 ng/mL with a mean of 31.18 ± 9.67 ng/mL. Females considered non-pregnant (n = 8) had serum progesterone levels of 0.1 to 0.71 ng/mL with a mean of 0.30 ± 0.24 ng/mL. While this study lacks pregnancy confirmation by ultrasound, there was a clear separation in serum progesterone levels between 0.71 ng/mL (highest presumed non-pregnant whale) and 10.85 ng/mL (lowest presumed pregnant whale) supporting the use of elevated serum values as diagnostic of pregnancy (Table 1). Serum progesterone values from pregnant animals sampled in May were found to be greater than those sampled in August/September (Table 2, *P* = 0.0318, Mann-Whitney U test). Five of the eight females captured in May were determined to be pregnant by serum progesterone analysis, four of the five levels were reported as > 40 ng/mL, one was lower at 16.89 ng/mL. Pregnant females (n = 8) captured in August/September had a mean of 28.55 ± 8.88 ng/mL of serum progesterone. The smallest pregnant female was 2.87 m in length (Figure 1), which corresponds to an estimate age of 9 years (Suydam, 2009, Figure 2–10). The four females smaller than 2.87 m in length were estimated to be 8 years old and based on serum and blubber progesterone levels, none of these animals were pregnant.

**Figure 2:**
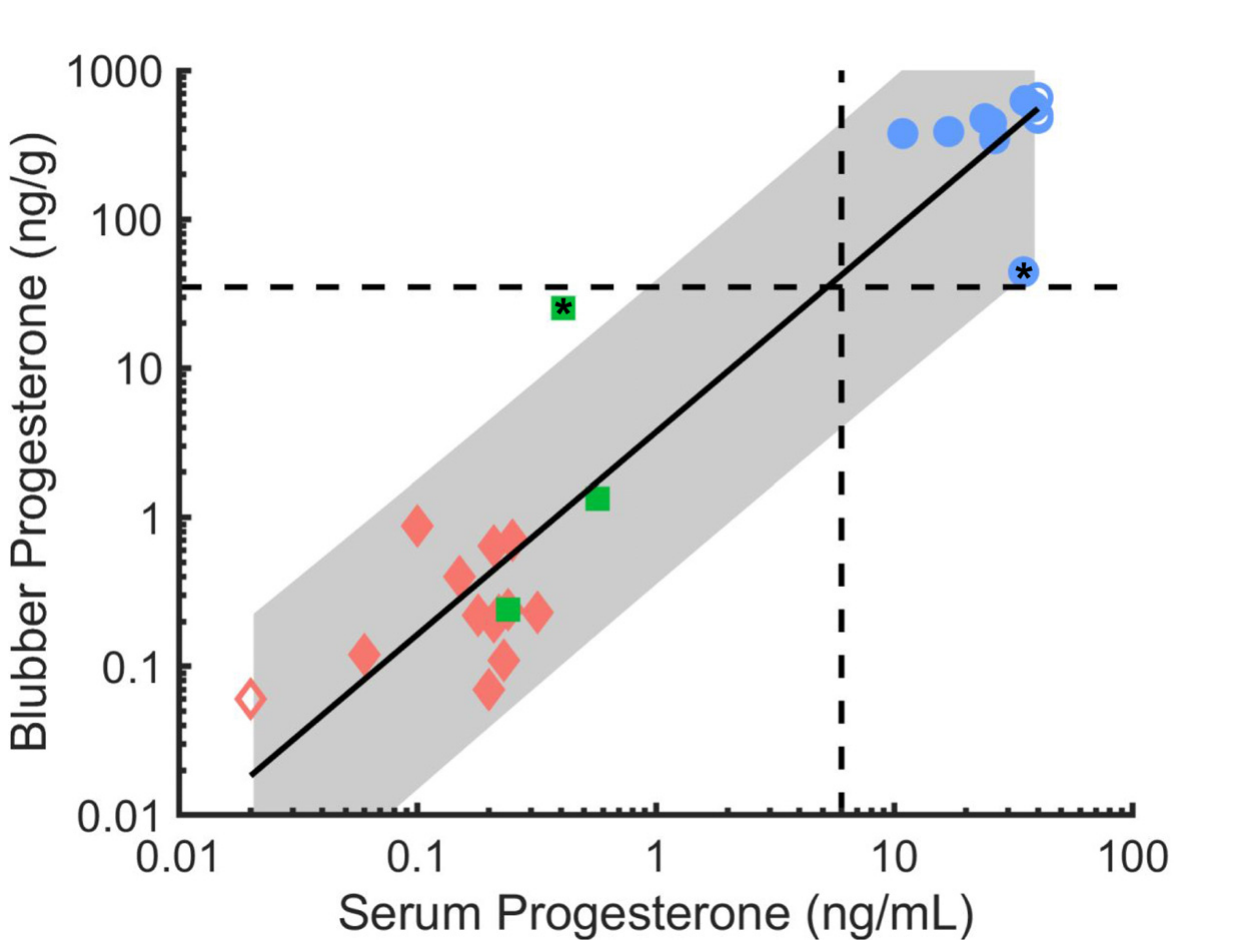
Blubber and serum progesterone concentrations (log scale) in beluga whales sampled in Bristol Bay (2008–2014) for males (red diamonds, n = 13), non-pregnant females (green squares, n = 3), and pregnant females (blue circles, n = 11). Belugas with values < 0.02 ng/mL and > 40 ng/mL were assigned values of 0.02 ng/mL (n = 6) and 40 ng/mL (n = 4) respectively (and depicted with open symbols). The two females with intermediate blubber values (with ^*^ in symbol) were assigned pregnancy status based on serum levels. Dashed lines divide pregnant and non-pregnant females based on progesterone levels, > 6.0 ng/mL in serum and > 35 ng/g in blubber. The linear regression equation is log10(y) = 1.357^*^log10(x) + 0.5695. The 95% CI, shown shaded in grey, for the coefficient is (1.179, 1.535) and for the intercept is (0.3698, 0.7693). The R^2^ is 0.91. The linear form of the equation is y = 3.7111^*^x^1.357^, with 95% CIs of (2.3431, 5.8790) and (1.179, 1.535), respectively.

### Blubber results

Twenty-seven beluga whales (14 females, 13 males) had blubber and serum analyzed for progesterone. The summary statistics for blubber values are presented in Table 1. Blubber and serum progesterone matrices showed strong agreement regarding progesterone levels (Figure 2). The regression equation is log_10_(blubber progesterone (ng/g)) = 1.357^*^log_10_(serum progesterone (ng/mL)) + 0.5695. The 95% CI for the coefficient is 1.179 to 1.535 and for the intercept is 0.3698 to 0.7693. The *R*^2^ is 0.91 (*P* < 0.00001). Males (n = 13) had low mean levels of progesterone in both blubber (0.32 ng/g) and serum (0.12 ng/mL). Two females also had low mean levels of progesterone in both blubber (μ = 0.79 ng/g) and serum (0.30 ng/mL) consistent with not being pregnant. Two other females (14%) had blubber with intermediate levels of progesterone, approximately one magnitude lower than the presumed pregnant and one magnitude higher than the presumed non-pregnant animals, including males (Figure 2). One female, with a blubber progesterone level of 44.30 ng/g had a serum level suggestive of pregnancy (34.83 ng/mL). The other female, with a lower blubber progesterone level of 25.37 ng/g, had a low serum level consistent with not being pregnant (0.41 ng/mL). Mean blubber progesterone concentration in pregnant females was 485.53 ± 104.52 ng/g (n = 10), excluding the females with intermediate blubber progesterone (Table 1). There was no significant difference in blubber progesterone between seasons for pregnant females which had a mean of 505.04 ± 111.91 ng/g of blubber progesterone in May (n = 4) and 472.52 ± 107.9 ng/g in August/September (n = 6) (Table 2).

## Discussion

While confirmation of pregnancy with ultrasound was not possible in this study, serum progesterone values clustered in statistically distinct groups to support using elevated values as diagnostic of pregnancy. Most females tested (13 of 21, 57.1%) were considered pregnant. Because gestation in beluga whales is longer than a year and pregnancy stage was not determined in our study, this percentage likely includes females that became pregnant the same year as being sampled (early gestation) and females that became pregnant the previous year (near term). Our percent pregnant is similar to that at Point Lay, Alaska (56%), which also included mature females in both early and late pregnancy and is higher than the approximately 33% reported for mature females in studies that can account for stage of pregnancy separately (Burns and Seaman, 1986; Suydam, 2009).

Longitudinal studies of beluga whales have shown serum progesterone peaks in early pregnancy (range, 60–66 ng/mL), decreases in mid-gestation (to 7–24 ng/mL) and decreases further in late pregnancy (to 6–9 ng/mL) (Calle *et al.*, 1993). In wild populations, early and late pregnancy stages overlap in the spring with mid-pregnancy occurring in the fall and winter. In this study, there was temporal clustering of serum progesterone levels that may reflect the stage of pregnancy. Of the values observed in May, the very high, > 40 ng/mL, would be consistent with early pregnancy while the lower value seen in one female (16.89 ng/mL), sampled in 2008, would be consistent with late pregnancy. The serum values seen in September were intermediate and consistent with mid-gestation. Determining the stage of pregnancy using just progesterone is imprecise but potentially can be improved by using testosterone or other hormones that have different temporal patterns throughout pregnancy as has been demonstrated for serum in bottlenose dolphins (Robeck *et al.*, 2018).

The smallest pregnant female in our study was 2.87 m in length, which corresponds to an estimated age of 9 years using data for the eastern Chukchi Sea stock (Suydam, 2009; Figure 2–10). The four smaller females, estimated by length to be 8 years old, were not pregnant even though half were likely sexually mature given their size (Suydam, 2009).

In general, there was distinct clustering and separation of high and low levels of progesterone in blubber. Additionally, there was agreement in paired serum and blubber tests with presumed pregnant female beluga whales having notably higher levels of progesterone in both tests compared with very low levels for males and presumed non-pregnant females. This method has been validated for several delphinid species, including *D. capensis, S. attenuata, S. longirostris* and *P. dalli*. Mean blubber progesterone levels in these species were 164 times higher in pregnant than non-pregnant females (Trego *et al.*, 2013). In *T. truncatus* and *G. melas* where pregnancy was confirmed by later photo identification of females with calves, progesterone was nine times higher than that found in non-pregnant females (Pérez *et al.*, 2011). Similarly, progesterone in confirmed pregnant minke whales was nearly 60 times greater than non-pregnant females (Mansour *et al.*, 2002), and levels in pregnant bowhead whales were orders of magnitude greater than non-pregnant females (Kellar *et al.*, 2013a). Except for the two intermediate values, mean blubber progesterone concentrations in this study were 600 times greater in pregnant females than in males and non-pregnant females. This difference is of a similar magnitude to dichotomous progesterone levels described in pregnant and non-pregnant humpback and bowhead whales (Clark *et al.*, 2016; Kellar *et al.*, 2013a; Pallin *et al.*, 2018a).

The blubber progesterone levels in two females (14% of females tested) were of an intermediate level. The higher blubber value corresponded with a higher serum value consistent with pregnancy and the lower blubber value corresponded with a lower serum value consistent with not being pregnant. Using the serum results as supporting information to assign pregnancy status produces non-overlapping groupings of the progesterone levels in blubber between non-pregnant (<35 ng/g) and pregnant (>35 ng/g) animals. However, the two intermediate blubber values were close enough to each other (25.37, 44.30 ng/g) that there would be increasing uncertainty when assessing pregnancy status the closer an individual's blubber progesterone concentration is to 35 ng/g if relying on blubber alone. Other supporting information (e.g. life history, photogrammetry, other hormone information) could aid in the evaluation of such cases. Past research comparing serum and blubber concentrations of progesterone in bowhead whales found a significant positive relationship between the two matrices and suggested that serum progesterone rises earlier in pregnancy than blubber progesterone (Kellar *et al.*, 2013a). When females are sampled shortly after conception, embryonic loss, parturition or abortion, the level of progesterone in blubber may be in transition, which may account for the intermediate values. However, results from this study do not support identifying the stage of pregnancy using blubber progesterone. While this may be a function of our small sample size, other studies also have not found evidence of a relationship between blubber hormone levels and gestational time (Kellar *et al.*, 2006). As with serological testing, examining blubber progesterone in combination with testosterone or other hormones may help determine the stage of pregnancy and resolve the status of females with intermediate levels of blubber progesterone.

Sampling additional live beluga whales and analyzing paired serum-blubber samples for progesterone, in combination with ultrasound confirmation of pregnancy status, and testing for additional hormones are needed to better understand the dynamics of blubber progesterone throughout pregnancy. Testing blubber of fresh dead animals in combination with morphological information about any fetus would also contribute to this knowledge. Nevertheless, for most cases, pregnancy status of beluga whales can be determined from blubber progesterone levels alone. The exception is for intermediate blubber progesterone levels for which additional information is necessary. Progesterone levels in blubber of beluga whales provide a valuable tool to determine reproduction dynamics of populations that cannot be captured for examination.
